# The Cellular Tumor Immune Microenvironment of Childhood Solid Cancers: Informing More Effective Immunotherapies

**DOI:** 10.3390/cancers14092177

**Published:** 2022-04-27

**Authors:** Malcolm Holterhus, Bianca Altvater, Sareetha Kailayangiri, Claudia Rossig

**Affiliations:** Department of Pediatric Hematology and Oncology, University Children’s Hospital Muenster, 48149 Muenster, Germany; malcolm.holterhus@ukmuenster.de (M.H.); bianca.altvater@ukmuenster.de (B.A.); sareetha.kailayangiri@ukmuenster.de (S.K.)

**Keywords:** cellular immunotherapy, tumor microenvironment, tumor-associated macrophages

## Abstract

**Simple Summary:**

Cellular immunotherapy has emerged as a novel treatment modality of cancer but is largely inefficient in solid cancers of childhood and adolescence. Besides tumor cells, solid cancers contain various bystander cell populations that can suppress immune responses and prevent the action of therapeutic effector cells within the tumor niche. This review summarizes current insights into the types of cells with an immunosuppressive function in the cellular microenvironment of common childhood cancers, along with novel approaches to overcome these barriers to enable effective immunotherapies.

**Abstract:**

Common pediatric solid cancers fail to respond to standard immuno-oncology agents relying on preexisting adaptive antitumor immune responses. The adoptive transfer of tumor-antigen specific T cells, such as CAR-gene modified T cells, is an attractive strategy, but its efficacy has been limited. Evidence is accumulating that local barriers in the tumor microenvironment prevent the infiltration of T cells and impede therapeutic immune responses. A thorough understanding of the components of the functional compartment of the tumor microenvironment and their interaction could inform effective combination therapies and novel engineered therapeutics, driving immunotherapy towards its full potential in pediatric patients. This review summarizes current knowledge on the cellular composition and significance of the tumor microenvironment in common extracranial solid cancers of childhood and adolescence, such as embryonal tumors and bone and soft tissue sarcomas, with a focus on myeloid cell populations that are often present in abundance in these tumors. Strategies to (co)target immunosuppressive myeloid cell populations with pharmacological anticancer agents and with selective antagonists are presented, as well as novel concepts aiming to employ myeloid cells to cooperate with antitumor T cell responses.

## 1. Introduction

### 1.1. Pediatric Solid Cancers: Resistance to T-Cell Based Immunotherapeutics

T cells can provide durable immune control of cancer. This is impressively illustrated by the therapeutic capacity of agents that restore the capacity of tumor-associated T cells to reject tumor cells by blocking inhibitory immune checkpoints [1,2]. Chronic activation of T cells in the tumor microenvironment (TME) results in upregulation of an inhibitory receptor, programmed cell death protein 1 (PD-1), which interacts with programmed death-ligand 1 (PD-L1) on tumor cells and/or bystander cells for functional suppression and exhaustion [3,4]. The antibody blockade of PD-1 receptor-mediated signals can achieve and maintain remissions in a variety of previously incurable cancers, such as metastatic melanoma, lung cancer, or renal cancer [5,6,7,8,9]. By contrast, pediatric cancers are not responsive to immune checkpoint blockades [10,11,12,13], with the rare exceptions of Hodgkin lymphoma and solid tumors arising on the basis of constitutional mismatch repair deficiencies [10,14]. Among several hundred pediatric patients treated in phase I/II studies, antibody inhibitors of PD-1 or PD-L1 did not have activity against any of the common solid cancers occurring in children and adolescents, such as embryonal cancers or bone and soft tissue sarcomas [15].

The failure of the therapeutic principle of immune checkpoint blockade can be explained by its prerequisites, along with typical features of pediatric cancers. The immune control of tumors in response to checkpoint inhibitors relies on the presence of tumor-associated antigens recognized as foreign by autologous T cells. Somatic gene mutations in tumors create neoantigens, some of which are capable of driving cytolytic T cell responses [16,17,18,19,20]. Accordingly, high tumor mutational burden is associated with the presence of tumor-infiltrating T cells [21] and with a clinical benefit to the checkpoint inhibitor blockade [16,22,23]. Systematic studies in adults have revealed that the immune contexture of a tumor, defined by the types, location, and density of infiltrating T cells, predicts the effective spontaneous or therapeutic immune response against cancer [24]. Based on digital immunohistochemistry analysis of paraffin sections, Jerome Galon’s group in France has established a clinical scoring system which reflects the abundance of memory T cells and cytotoxic T cells in the tumor center and in the invasive margin [25]. The immunoscore of individual tumors was strongly associated with the outcome and had independent prognostic value even superior to classical tumor staging criteria in various solid cancers in adults [26]. Pediatric tumors are driven by the epigenetic deregulation of gene transcription, rather than the accumulation of somatic gene mutations [27,28,29]. Consequently, they have only few neoantigens and are ignored by the T cell immune system. While Galon´s immunoscore has never been applied to pediatric cancers, low numbers of T cells and rare expression of PD-L1 has been found in various independent studies [30,31]. Thus, pediatric cancers are T-cell deficient tumors and thus, highly challenging targets for immunotherapies.

### 1.2. Composition of the Tumor Microenvironment: Key to Effective Immunotherapies

Despite the reproducible associations between tumor mutational load, spontaneous T cell infiltration, and response to immune checkpoint inhibitors, these factors alone do not explain immunogenicity or the lack thereof in solid tumors. A relatively low mutational load does not preclude activity of checkpoint inhibitor therapy in adult cancer patients [32,33], and in hematological cancers, rare neoepitopes, and even peptides from normal non-mutated proteins, can allow T cells to mount effective antitumor responses [34,35]. However, neuroblastoma (NBL) remains insensitive to checkpoint inhibition at relapse [10,11,12,13], despite an increasing burden of somatic gene mutations along with disease progression [36]. Attempts to replace pre-existing adaptive immunity by adoptive transfer of ex vivo generated tumor-antigen specific T cells, e.g., by gene-engineering with chimeric antigen receptors (CARs), also failed to produce and sustain objective responses [37,38,39,40,41]. Thus, beyond the scarcity of potential rejection antigens in tumor cells, further barriers must exist in pediatric solid tumors that prevent the emergence of natural adaptive immune responses and block the activity of adoptively transferred antitumor T cells. 

Tumor cells in solid cancers grow within a network of stroma with blood vessels, lymphatic vessels, and bystander cells. Cell infiltrates in the TME of T-cell deficient tumors are often dominated by cells of myeloid progeny [42,43,44], which can have strong immunosuppressive properties and act as barriers against therapeutic T cell responses [45,46,47,48,49]. To design immune-based therapies that effectively target pediatric tumors in their anatomical and functional niches, a detailed understanding of the nature and function of these cell populations and their interactions is needed. This review starts by introducing the major myeloid cell components of human cancers, along with an overview of state-of-the art tools to characterize the TME. We then summarize knowledge on the roles of myeloid tumor cell components in pediatric cancers and suggest strategies to bypass or employ the TME for novel combination therapies.

## 2. Myeloid Cell Components of the Immune Microenvironment of Solid Tumors

**Figure 1:**
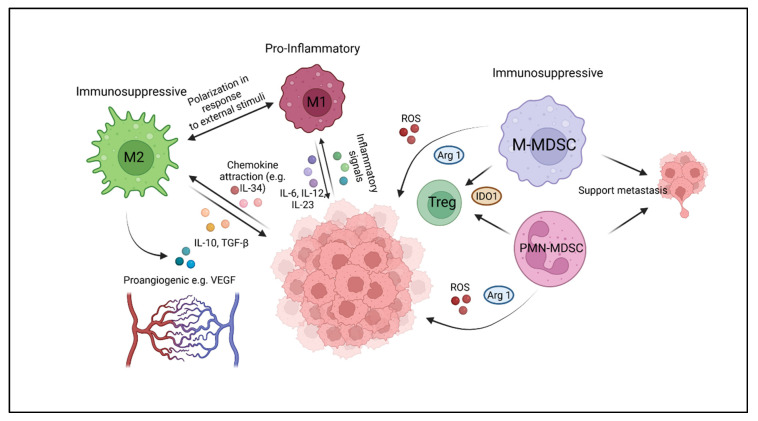
Myeloid cell components of the tumor microenvironment and their interaction with tumor cells and alternative bystander cells. Arg1: arginase 1; IDO1: indoleamine 2,3-dioxygenase 1; IL: interleukin; M1: M1-polarized tumor associated macrophage; M2: M2-polarized tumor associated macrophage; M-MDSC: monocytic myeloid-derived suppressor cell; PMN-MDSC: polymorphonuclear myeloid-derived suppressor cell; ROS: reactive oxygen species; TGF-β: transforming growth factor beta; Treg: regulatory T-cell; VEGF: vascular endothelial growth factor. The Figure was created with BioRender, accessed on 3 March 2022.

Various myeloid cell populations can be recruited to the sites of solid tumors, where they exploit physiological programs involved in wound healing and tissue protection to promote local tumor growth and metastatic spread. Here, we briefly review the key populations, tumor-associated macrophages (TAM), and myeloid-derived suppressor cells (MDSC) (Figure 1). Tumor-associated neutrophils (TAN) have been identified as an additional inhibitory cell population in human tumors [50]) but will not be included into this review, since knowledge of their role in pediatric cancers is scarce.

### 2.1. Tumor-Associated Macrophages (TAM)

Macrophages are immune cells of mononuclear origin with a phagocytic function. They have the ability to infiltrate into almost every tissue. On the basis of their differentiation status, marker expression, and functional roles, macrophages are classified into pro- and anti-inflammatory M1 and M2 subtypes, respectively (reviewed in [51,52]). M1-like polarization is induced by activation of macrophages with interferon-γ (IFN-γ), Toll-like receptors or (bacterial) lipopolysaccharides. M1-polarized macrophages have critical roles in host defense. They secrete pro-inflammatory cytokines and can lyse cells directly via reactive oxygen species (ROS) and nitrogen oxide, or through antibody-dependent cell-mediated cytotoxicity (ADCC). Their phenotype in humans is characterized by the expression of CD68, CD80, and CCR7. Human M2-like macrophages arise from alternative activation and express CD68, along with CD163. Their physiological role is to protect tissues from destructive immune responses and to promote tissue and vessel remodeling by secretion of immunosuppressive, proangiogenic, and growth factors. In response to stimuli from the tissue environment, macrophages can adjust their functional states by epigenetic transcriptional regulation to represent diverse subtypes beyond the binary distinction of M1 versus M2, which predominantly kill or repair [53].

TAM are actively recruited into tumors from their circulating monocyte progenitors by inflammatory signals provided by cancer cells. They promote tumor growth and negatively affect T cell metabolism and effector function [54]. Even immunodeficient mouse models can have intact (murine) macrophage populations [55], which can enhance the progression of human tumor xenografts and protect them against antitumor immunotherapies [56,57]. The current definition of TAM continues to distinguish M1- and M2-like phenotypes, even though this model fails to reflect the plasticity of polarization under in vivo conditions [51]. This seems justified by the association of M2-polarized TAM with poor prognosis in various cancers [58,59,60,61,62], whereas the predominance of M1-like TAM predicts favorable outcomes [63,64,65]. Efforts are under way to understand in more detail the roles and mechanisms of individual functional subtypes in preventing or supporting protective and therapeutic immune responses in human cancers.

### 2.2. Myeloid-Derived Suppressor Cells (MDSC)

The term MDSC defines a heterogeneous population of immature myeloid cells with either monocyte- or granulocyte-like differentiation and a strong ability to suppress T cell function. MDSC have an important role in maternal-fetal tolerance, and their transient presence during the first weeks of life can protect the newborn by providing an antimicrobial function [66]. Beyond neonatal age and under physiological conditions, MDSC are largely absent. Their predominant role is to be recruited to sites of chronic infection and malignant tumor growth, where they exert a potent immunosuppressive function (reviewed in [67]). In murine tumor models, MDSC were found to originate from circulating hematopoietic progenitor cells that are released by the bone marrow in response to signals from the distant tumor site, then differentiate into MDSC in the tumor niche [68].

Two major subtypes of MDSC are described in humans: neutrophil-like granulocytic MDSC, also termed polymorphonuclear (PMN-)MDSC, and monocytic (M-)MDSC. M-MDSC can differentiate into TAMs in tumors where this type typically predominates [69]. The phenotypic characterization of MDSC in humans relies on the coexpression of CD11b and CD33, in the absence of lineage markers, and of HLA-DR, along with CD15, CD66b, and a lack of CD14 in PMN-MDSC, or with CD14 and CD16low in the absence of CD66b and CD15 in M-MDSC [67]. Mechanisms by which MDSC dampens T cell responses include the depletion of nutrients, such as arginine, by the production of arginase I [70] and the production of suppressive ROS [71]. Beyond tissue-resident MDSC, a circulating type of MDSC that shares features of fibrocytes, a mesenchymal cell population with inflammatory and pro-angiogenic functions, was identified in the peripheral blood of cancer patients [72]. Fibrocystic MDSC can exert a potent systemic T cell suppressive function by the release of the immune-suppressive enzyme indoleamine 2,3-dioxygenase 1 (IDO1).

The phenotypic plasticity of human MDSC complicates their identification and categorization in human cancer tissues, as well as in functional studies. Therefore, insights into the biology and relevance of this cell population largely rely on murine models. In a classic experiment, in vivo depletion of MDSC alone was sufficient to protect mice against tumor challenge [73], supporting their key role in immune evasion. For technical reasons, associations of MDSC populations and prognosis in cancer patients are also largely limited to studies in peripheral blood. Blood counts of both PMN-MDSC and M-MDSC showed predictive value for the prognosis and response to checkpoint inhibitor therapy in adult patients with solid tumors [74,75]. Overall, MDSC, with their potent immunosuppressive capacity, are prime suspects for protecting human tumors against adaptive immune responses and thus, are candidate targets for therapeutic intervention.

## 3. Tools to Decipher Cellular Components of Tissues

Along with an increasing awareness of the importance of understanding local mechanisms of tumor immunogenicity and immune escape, advanced technologies have been developed for the analysis of the TME. Cell populations in tumor tissues can be identified and characterized by (1) antibody detection of specific cellular markers, (2) analysis of gene expression, or (3) unbiased screening of proteins and other molecules.

### 3.1. Antibody-Based Analysis of Tissues

While classical antibody-based marking of cells on tissue sections by **immunohistochemistry** (IHC) or **immunofluorescence** (IF) is limited by the number of antibodies applicable on an individual slide, multiplex immunofluorescence staining with **multispectral imaging** allows the simultaneously detection of up to eight different immune markers on a single tissue section [76,77,78]. **Cyclic immunofluorescence imaging platforms** can identify more than 100 antigens simultaneously in a single biological sample. This is achieved in a fully automated process in which a tissue section undergoes repeated cycles of staining with fluorochrome-labeled antibodies, fluorescence imaging, and signal erasure [79,80,81].

Multiplex imaging technologies that rely on antibody staining not only discriminate and quantify individual cell populations, but also provide information on the spatial relationships between individual immune cells and tumor cells. Recent developments address the heterogeneity of the TME across tumors which is not adequately reflected by standard two-dimensional analysis of tumor tissue sections. Three-dimensional spatial imaging can be achieved by forming a hydrogel matrix, using the CLARITY method, to generate a transparent and structurally intact tissue [82]. Originally developed for the field of neuroscience, CLARITY processing of tumor tissues was also found to adequately visualize the TME in 3D [83].

Advances in multicolor flow cytometry beyond 12 colors now also allows for detailed characterization of the heterogeneous cellular components of the TME [84], although at the expense of the relationships with neighboring cells and the overall context of tissue architecture. Individual cells from single-cell suspensions of disintegrated tumor tissues can be analyzed with this technology in a quantitative and high-throughput manner, with high accuracy.

### 3.2. Gene Expression Analysis of the TME

Cells with various phenotypes and functions can be distinguished on the basis of their gene expression. In tissues, this is achieved by **RNA in situ hybridization (RNA ISH)**. Novel RNA ISH techniques with enhanced sensitivities allow for the detection of even single mRNA transcripts using standard microscopy. Combined with IHC/IF, ultrasensitive RNA ISH provides detailed transcriptomic information on specific cell populations in defined cellular localizations [85,86].

**Computational transcriptomic tools** such as CIBERSORT [44,87], Microenvironment Cell Populations-counter (MCP-counter) [88] or TIMER2.0 [89] use gene expression data to estimate the abundance of individual immune cell populations among the cellular composition of the TME and infer immune functions. More extensive **immunogenomics profiling** with **single-cell technologies** now allow us to gain detailed information beyond enumerating cell subpopulations, including aspects of cell signaling and function, along with somatic DNA alterations, neoantigen prediction, and TCR repertoire analysis [90,91,92]. While these advanced genomic and transcriptomic analysis tools provide extensive and detailed information, their limitation is the lack of spatial information.

To obtain comprehensive insights into ongoing immune interactions in cancers and to understand relationships between factors of the tumor and TME with immunogenicity or the lack thereof, a single strategy will not be sufficient. Investigators have now started integrating immunogenomic methods with antibody-based analysis in parallel studies of cell suspensions at the single-cell level, along with intact tumor tissue [91,92].

### 3.3. Unbiased Molecular Profiling

Mass spectrometry allows for the identification of thousands of proteins and other molecules by ionization, without the use of antibodies and without a priori knowledge of candidates. Using matrix-assisted laser desorption/ionization (MALDI) for ionization, mass spectrometry can be directly applied to the in situ analysis of tissues, preserving spatial information [93]. This technology, **MALDI imaging mass spectrometry (MALDI-IMS),** is used as a tool to discover novel biomarkers and molecular targets in individual cancers, and more recently, to visualize immune-related factors of the TME [94].

## 4. Composition of the Immune Microenvironment of Pediatric Cancers

This review will focus on the typical extracranial tumors of childhood and adolescence, including NBL and bone and soft tissue sarcomas. A pilot study of immune cell infiltration in pediatric solid tumors was performed by Michael Lotze´s group in 2006, using standard IHC analysis of 27 pediatric tumor samples of all types [42]. Compared to carcinomas in the adult population, pediatric tumors showed significantly higher infiltration by CD68+ macrophages. T cells were present in low numbers, and dendritic cells were largely absent. More recently, the immunogenomic and transcriptomic analysis of various relapsed or refractory solid tumors from 202 pediatric cancer patients with the application of an immunoscore revealed a general absence of immune infiltration [36]. The following paragraph summarizes the knowledge on individual disease entities, which is also illustrated in Table 1.

### 4.1. Neuroblastoma (NBL)

Lymphocyte infiltrates in NBL have been the subjects of a large number of studies (recently reviewed in [95]). While a considerable variability of T cell subtypes and infiltration densities between and even within individual tumors were reported, higher T cell density was a favorable prognostic factor in most studies. In one of the largest and most detailed investigations, deep RNA sequencing of pretreatment tumors from 150 NBL patients, with an additional validation cohort of 190 tumors, identified a gene expression signature of cytotoxic tumor-infiltrating lymphocytes that was associated with MYCN non-amplified tumors and with improved outcomes [96].

Substantially less focus in this cancer has been on myeloid bystander cells. Among 71 NBL tumors, Bob Seeger´s group found significantly higher numbers of infiltrating CD163+ (M2-like) TAM in samples from patients with metastatic compared with locoregional disease [43]. An inflammation-associated gene signature of high-risk disease emerging from the same patient population included various genes representing macrophages and their polarization, further supporting a relevant contribution of TAM to aggressive tumor behavior. A correlation between higher numbers of CD163-expressing TAM and metastatic disease was reproduced in an independent IHC study on 41 NBL samples [97].

Functional evidence was generated in syngeneic mouse models of NBL. Spontaneously arising tumors in a transgenic mouse model demonstrated a transition from a TME dominated by CD8+ T cells in early neoplastic lesions towards enrichment of both MDSC and TAM, along with M1 to M2 transition, during tumor progression [98]. An alternative investigation in an immune-competent NBL mouse model found an enrichment of MDSCs in spleens, bone marrow, and peripheral blood in tumor-bearing compared to tumor-free animals [99]. Moreover, monocytic MDSCs in NBL-bearing mice expressed major mediators of MDSC-driven immunosuppression, including arginase-1, ROS, and TGF-β [99]. Again in a transgenic mouse model of NBL, cross-talk between tumor cells and myeloid cells was found to promote tumor progression. Macrophages in the presence of tumors were polarized towards the secretion of cytokines that in turn affected arginine metabolism in NBL [100].

Overall, IHC and RNA expression data in human NBL, along with findings in murine models, associate the presence of myeloid cell populations and/or poor T cell infiltrates with advanced and aggressive disease. Whereas infiltrating T cells may be involved in protective immune responses in NBL, M2-polarized TAM could be relevant players in tumor immune suppression.

### 4.2. Osteosarcoma (OS)

Various studies of human OS tissue samples by IHC or transcriptomic analysis have all identified TAM as a major infiltrating immune cell population [101,102,103,104,105,106,107]. Data about their prognostic value and their association with tumor aggressiveness and metastasis are inconsistent. In tumors from treatment-naive patients, high numbers of overall TAM, defined by gene expression signatures (*n* = 53) and validated by IHC (79 additional samples), were associated with a superior prognosis [101]. More recent studies differentiated between M1- and M2-polarized macrophage phenotypes. Among 11 OS tumor samples analyzed by RNA single-cell sequencing, the majority of TAM had M2-like gene expression [103]. In a uniformly treated cohort of 124 patients, high numbers of CD163+ (M2-like) TAM in pretherapeutic biopsies by IHC were associated with a favorable outcome, defined by longer metastasis progression-free survival [102]. However, this association was no longer observed in a follow-up study, in which patients received zoledronic acid, an inhibitor of osteoclast function, as part of their adjuvant treatment regimen [108]. Another study, based on pretherapeutic samples from 22 localized and 28 metastatic OS tumors, found M1-polarized macrophages to be associated with favorable (non-metastatic) disease [106].

The predictive significance of TAM subpopulations may be affected by neoadjuvant chemotherapy. OS tissues from 68 patients obtained at the time of definitive surgery revealed higher CD68+ TAM densities in tumors from patients with lung metastases versus those with localized tumors [104]. A comparative study directly addressed the effects of chemotherapy on immune cell populations in the TME by IHC/IF analysis of matched biopsy and surgical samples of 27 OS patients. An increase in CD8+ T cells coexpressing PD-L1 was observed following chemotherapy, without significant changes in the density of TAM, but along with a decrease in MDSC (HLA-DR-/CD33+) [109]. TAM infiltration may further vary with different disease sites. In paired samples from individual patients (*n* = 18), lung metastases contained higher numbers of TAM than (pretreated) primary tumors [104], and RNA single-cell sequencing analysis of two lung metastasis samples revealed infiltration by macrophages with a gene expression profile of alveolar, or tissue resident macrophages [103].

Together, these correlative studies suggest that the recruitment of macrophages and/or infiltration with tissue resident TAM are involved in aggressive and metastatic tumor growth in OS, with high context-dependent variability.

Functional in vivo studies in mouse models support a growth-promoting and immunosuppressive role of M2-polarized TAM in OS. In a syngeneic OS mouse model, macrophages coinjected along with tumor cells enhanced metastasis to the lungs, with enrichment of M2-like TAM in the TME of established metastases [110]. The inhibition of TAM polarization in this model reduced the number of pulmonary metastatic nodes. M2 macrophage polarization was also seen in immunodeficient xenograft models at the sites of implanted tumors or lung metastases, and therapeutic elimination of TAM [111] or antiinflammatory treatment decreased tumor xenograft growth [101]. Moreover, in a murine xenograft model, tumor cells were found to actively recruit M2-like, arginase I-expressing TAM into OS tumors [112]. Kansara et al. discovered a functional connection between myeloid cell infiltration in OS and tumor growth when following up on a finding from genome-wide association studies in humans [113]. The gene locus for a glutamate receptor, glutamate metabotropic receptor 4 (GRM4), was linked to the susceptibility to OS. In a mouse model, GRM4 originated from monocyte-derived DCs in the TME and acted to drive tumor growth via proinflammatory IL-23.

While there is little doubt now that myeloid tumor-infiltrating cells significantly affect tumor growth in OS and are worth investigating as therapeutic targets, many aspects of their roles and crosstalk with tumor cells are not yet understood.

### 4.3. Ewing Sarcoma (EwS)

TAM are also a predominant cell population in EwS, and an exploratory analysis of human tumors, as well as experimental studies, suggest a role for TAM in disease progression.

One of the first studies in EwS found that a higher extent of CD68+ macrophage infiltration in 41 human EwS samples by IHC was associated with poorer overall survival [56]. Consistently, gene expression analysis and the enumeration of immune cell subpopulations by CIBERSORT, using data from 197 primary EwS tumor samples, found abundant macrophages, predominantly M2-polarized, and high M2 TAM infiltration was associated with poor outcome [114]. By contrast, high total T cells, along with low M2 TAM in a small patient population, was linked to favorable event-free survival.

Due to the lack of immunocompetent syngeneic mouse models for EwS [115], in vivo studies were typically performed in xenograft models studying human tumor cells along with murine myeloid cells. The depletion of murine TAM in a xenograft model using liposome-encapsulated clodronate significantly inhibited the development of human EwS [56]. Based on the observation that TAM was associated with greater microvascular density, the authors proposed a link between TAM recruitment to EwS and stimulation of angiogenesis, with a key role of vascular endothelial growth factor (VEGFR) secreted by EwS cells. A relevant role of TAM in human EwS xenograft growth was confirmed by alternative investigators. Treatment with an inhibitor of TAM-derived inflammatory mediators had potent antitumor activity in a metastatic EwS xenograft mouse model, substantially reducing the metastatic burden [116], and in vitro, the agent reduced tumor cell invasion and extravasation of EwS cells stimulated by M2-polarized macrophages [116].

In the search for molecular mechanisms regulating TAM recruitment and TAM-mediated tumor growth promotion, aberrant gene regulation downstream of the disease-defining chimeric transcription factor EWSR1-FLI1 was proposed. Indeed, the overexpression of a regulatory microRNA suppressed by EWSR1-FLI1, let-7a, was found to decrease macrophage infiltrations in EwS-xenografted tumors in vivo, and to reduce tumor aggressiveness, via modulation of the STAT3 signaling pathway [117].

Experimental evidence supports a high relevance of tumor-infiltrating immune-suppressive myeloid cells in the protection of pediatric bone sarcomas against adoptive T cell targeting. The growth of EwS and also OS xenografts induced the expansion of granulocytic murine MDSC in the blood, spleens, and tumors of NSG mice, which was associated with a lack of antitumor activity of tumor-targeted CAR T cells, and MDSCs isolated from spleens of these mice suppressed CAR T cell proliferation in coculture experiments in vitro [57].

### 4.4. Rhabdomyosarcoma

In rhabdomyosarcoma (RMS), the most common soft tissue sarcoma in childhood, large comprehensive studies of the cellular components in the TME are lacking. Consistent with results in bone sarcomas, IHC and digital pathology analysis of tumor-infiltrating immune cells in 39 primary tumors revealed an overall low number of TIL, along with more abundant TAM [118]. Embryonal RMS (*n* = 20) had a higher degree of immune cell infiltration, including both T cells and TAM, and higher microvascular density compared to alveolar (*n* = 19) RMS. In a gene expression analysis of various soft tissue sarcomas, RMS had comparatively high CD8+ T cell infiltration [119]. Another gene expression analysis, in combination with IHC, compared the TME of RMS (*n* = 27 embryonal, *n* = 24 alveolar) with undifferentiated polymorphic sarcoma (UPS), a cancer of the adult population which can respond to checkpoint inhibition. The two tumor types had similar immune cell compositions, with predominant M2 macrophages and variable T cell infiltration; but, whereas T cells in UPS diffusely infiltrated the tumors, they clustered in tertiary lymphoid structures in both alveolar and embryonal RMS [120]. Thus, the spatial in situ distribution of TILs in tumors and potential trapping in niches within the TME could be important determinants of their ability to interact with tumor cells.

In an orthotopic murine model of rhabdomyosarcoma, granulocytic MDSCs were found to mediate strong local immunosuppression, limiting the efficacy of the immune checkpoint blockade [121]. MDSC infiltration into the TME in this model relied on interaction between the chemokine CXCR2 and its ligands. CXCR2 ligands are produced by pediatric OS and RMS cell lines and detected in the serum of pediatric sarcoma patients; thus, they are candidate attractants for MDSC recruitment into RMS [121].

Table 1 summarizes published findings on the composition of the TME in the individual tumor entities.

**Table 1:** Findings of immune infiltrating cells in pediatric extracranial solid tumors. TAM: tumor-associated macrophages; MDSC: myeloid-derived suppressor cells; Ref.: Reference; RNA seq: RNA sequencing; IHC: immunohistochemistry; Arg-1: arginase-1; BM: bone marrow; ROS: reactive oxygen species; TGF-β: transforming growth factor beta; GEP: gene expression profiling; MPFS: metastasis progression-free survival; IF: immunofluorescence; Fc: flow cytometry; aRMS: alveolar rhabdomyosarcoma; eRMS: embryonal rhabdomyosarcoma.

Neuroblastoma	Patients	Samples	Method	Findings/Impact on Survival		Ref.
Material				TAM	MDSC	
**Human studies**						
pretreated tumor	71 (IHC) 133 (RNA seq)	133 (RNA seq) 71 (IHC)	RNA seq, IHC	M2 high in metastatic disease		[43]
pretreated tumor	41	41	IHC	M2 associated with metastatic disease		[97]
**Mouse models**						
syngeneic				TAM accumulate during progression, shift from M1 to M2	MDSC accumulate during disease progression	[98]
syngeneic					present in spleens, BM, blood, expressing Arg-1, ROS, TGF-β	[99]
transgenic				Crosstalk with tumor cells polarizes macrophages for tumor progression		[100]
**Osteosarcoma**						
**Human studies**						
primary tumors/ metastases	53(GEP), 117(IHC)	53(GEP), 174(IHC)	GEP/IHC	M1 and M2 associated with superior OS		[101]
primary tumors	124	124	IHC	M1 associated with superior OS, MPFS		[102]
primary tumor/ metastasis	50	22 localized 28 metastasis	IHC	TAM associated with superior OS, M1 predominant in non-metastatic disease		[106]
pre-/post treatment (matched)	27	54	IHC/IF	unchanged by chemotherapy	reduced after chemotherapy	[109]
**Mouse models**						
syngeneic			IHC/IF	M2 associated with metastasis		[110]
xenograft			Fc	M2 associated with tumor growth		[111]
xenograft				M2 recruited by tumor		[112]
**Ewing sarcoma**						
**Human studies**						
primary tumor pretreatment	41	41	IHC	high M1 associated with lower OS		[56]
primary tumor pre-treatment	197	197	GEP	high M2 associated with poor outcome		[114]
**Mouse models**						
xenograft			IHC	TAM stimulate angiogenesis		[56]
xenograft			IHC	TAM inhibition reduces metastatic burden		[116]
xenograft				TAM negatively regulated by miRNA let-7a		[117]
**Rhabdomyosarcoma**						
**Human studies**						
primary tumors	39	20 aRMS 19 eRMS	IHC	high TAM infiltration		[118]
primary tumors	51	24 aRMS 27 eRMS	IHC/GEP	high M2 infiltration		[120]
**Mouse models**						
orthotopic			Fc		CXCR2-mediated tumor infiltration, promote local immunosuppression	[121]
	

## 5. Strategies to Modify the TME for Effective Immunotherapies

### 5.1. Pharmacological Modification of the TME

**Figure 2:**
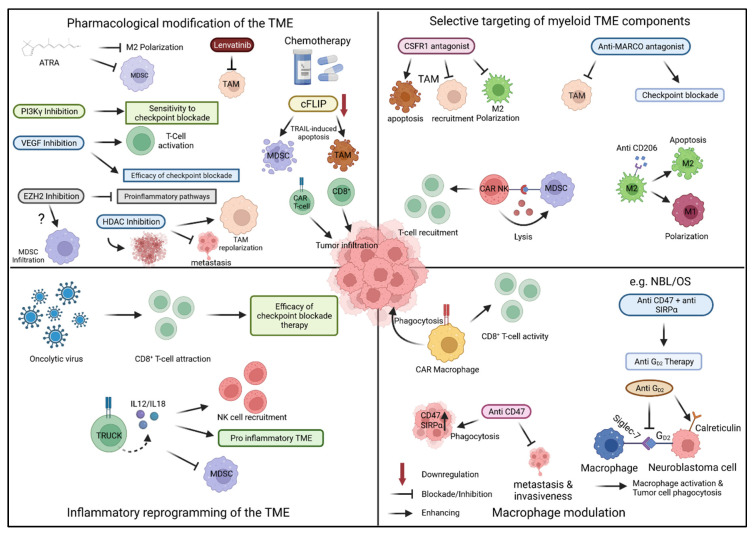
**Strategies to modify the TME for enhanced antitumor activity of T-cell based immunotherapy.** ATRA: all-trans retinoic acid; MDSC: myeloid-derived suppressor cell; TAM: tumor associated macrophage; cFLIP: cellular FLICE (FADD-like IL-1β-converting enzyme)-inhibitory protein; PI3Kγ: phosphoinositide3-kinase gamma; VEGF: vascular endothelial growth factor; EZH2: enhancer of zeste homolog 2; HDAC: histone deacetylase; CSFR1: colony stimulating factor 1 receptor; MARCO: macrophage receptor with collagenous structure; CAR NK: chimeric antigen receptor natural killer cell; TRUCK: T cells redirected for antigen-unrestricted cytokine-initiated killing; SIRPα: signal-regulatory protein alpha. The Figure was created with BioRender, accessed on 3 March 2022.

Knowledge of the types of tumor-associated cell populations that prevent protective immune responses in individual cancers can inform strategies to deplete these cells from tumor tissue or reduce or reverse their immunosuppressive function (Figure 2). Various agents used for cancer therapy, including standard cytotoxic agents and molecularly and epigenetically targeted agents, have been found to affect the composition of the TME by selective effects on tumor-associated myeloid cell populations. These agents are candidates for rational combination therapies with T-cell based immunotherapies.

#### 5.1.1. Targeting the TME by Standard Anti-Proliferative Chemotherapeutic Agents

Chemotherapy with standard anti-proliferative, cytotoxic agents can substantially affect the composition of the TME by eliminating myeloid cell subpopulations or reprogramming their function. In independent experiments performed in immunocompetent mouse tumor models, treatment with antiproliferative cytotoxic agents at low doses selectively decreased the number of MDSC in the spleens and in tumor beds [122,123,124,125]. The depletion of MDSC in these models was found to break immune tolerance and enhance the antitumor activity of adoptive T cell transfer [126]. Some evidence for the capacity of cytotoxic chemotherapy to alleviate the immunosuppressive effects of myeloid cell populations was also found in human cancer patients. In patients with advanced cervical cancer, treatment with carboplatin and paclitaxel reduced the number of circulating myeloid cells, and this was associated with the stimulation of stronger T cell reactivity to vaccination with a human papilloma virus peptide vaccine [127].

Individual cytotoxic agents substantially differ in their capacity to induce MDSC depletion. In murine models, gemcitabine and 5-fluorouracil (5-FU) were highly effective [123,124]. More recently, the DNA-binding cytotoxic agent trabectedin was identified as an agent with exceptionally strong effects on the non-tumor cell components of the TME. In a mouse tumor model, trabectedin rapidly reduced the number of circulating Ly6C^high^ monocytes, as well as TAM [125]. In two human sarcoma patients treated with trabectedin, monocytes decreased in the peripheral blood after each cycle, and a strong reduction in the density of TAM was found in post-therapeutic biopsies [125]. Trabectedin monotherapy in an immunocompetent OS mouse model increased infiltration of CD8+ T cells into tumors, associated with reduced numbers of TAM, though to a lesser degree than in other cancer models, and the combination of trabectedin with the PD-1 antagonist antibody showed synergism in controlling tumor progression [128].

The mechanisms of apoptotic cell death may determine the capacity of individual antiproliferative agents to contain immunosuppressive myeloid cell populations [122,129]. Trabectedin depletes the macrophages by activating tumor necrosis factor-related apoptosis-inducing ligand (TRAIL) receptors, with subsequent recruitment of caspase-8 and activation of the apoptotic cascade. TAM differentially express TRAIL receptors with intact signaling, whereas alternative cell populations are protected by expressing decoy receptors [125]. More recently, cellular FLICE (FADD-like IL-1β-converting enzyme)-inhibitory protein (cFLIP), the major player in TRAIL receptor-induced apoptosis, was found to have a key role in mediating macrophage depletion by antiproliferative cytotoxic agents [129]. Immunosuppressive monocytic myeloid cells rely on high and continuous levels of cFLIP for survival and thus, are highly vulnerable to cFLIP downregulation [130]. Among various agents, carboplatin, paclitaxel, irinotecan, gemcitabine, and 5-FU are strong downmodulators of cFLIP.

Rather than depleting TAM, cytotoxic agents can also modify their function towards actively supporting T cell infiltration. Srivastava et al. reported that the addition of oxaliplatin to cyclophosphamide in a mouse model of non-small cell lung cancer activated proinflammatory pathways and chemokine expression in TAM, resulting in improved recruitment of CAR T cells and more potent antitumor activity [131]. In a human patient with breast cancer, oxaliplatin/cyclophosphamide pretreatment followed by CAR T cell therapy also led to an increase in intratumoral CAR T cells, along with a decrease in M2-type macrophages.

An alternative clinically available agent known to affect the number and functions of MDSC is all-trans retinoic acid (ATRA), a regulator of cell differentiation. In murine tumor models, ATRA eliminated immature myeloid cells by maturation, thereby enhancing the T cell response to cancer vaccines [132]. This effect was reproduced in patients with metastatic renal cell carcinoma, in which ATRA at high plasma concentrations, together with subcutaneous interleukin-2 (IL-2), reduced the number of circulating myeloid suppressor cells and improved tetanus-toxoid-specific T cell responses [133]. In a mouse xenograft model of a pediatric cancer, EwS, ATRA reduced the numbers of MDSC in the peripheral blood, as well as their suppressive capacity, and the co-administration of ATRA with G_D2_-specific CAR T cells enhanced their antitumor activity [57]. In a syngeneic OS model, ATRA reduced OS tumor formation by blocking M2 polarization of TAM [134].

Thus, the well-informed integration of novel immunotherapies into the established backbones of pediatric cancer therapy could substantially improve their activity. 

#### 5.1.2. Targeting the TME by Molecularly Targeted Anticancer Agents

Signaling by tyrosine kinases is involved both in the proliferation of malignant tumor cells and in the tumor-supportive function of inflammatory cells in the TME. Consequently, clinical translation of treatment with molecularly targeted agents needs to take into account their impact on the TME, e.g., the inhibition of the Janus kinase (JAK) signal transducer and the activator of the transcription (STAT) pathway with ruxolitinib in a breast cancer model had direct antitumor effects, but at the same time, induced macrophage production of protumorigenic inflammatory mediators, leading to resistance [135]. 

Identification of signaling pathways that act as molecular drivers of the disease while negatively affecting immune cell components of the stroma could lead to synergistic blocking strategies. Myeloid cell recruitment to tumors and the production of inflammatory mediators was found to rely on signaling via the phosphatidyl-inositol 3 kinase γ isoform (PI3Kγ) in various mouse models [136,137]. Selective inhibition of PI3Kγ can prevent the migration of macrophages into tumors and promote repolarization toward the proinflammatory phenotype and function, restoring sensitivity to checkpoint inhibitors [137,138]. PI3Kγ inhibitors combined with PD-1 checkpoint antagonists are currently investigated in clinical studies. The multityrosine kinase inhibitor lenvatinib, which is investigated as an anticancer agent in pediatric OS [139,140], was also found to modulate the TME by decreasing the number of TAM, resulting in synergistic activity with PD-1 checkpoint inhibition in mouse models of hepatocellular and colorectal carcinomas [141].

A promising candidate for molecular targeting strategies focusing on the TME is vascular endothelial growth factor-A (VEGF) and its receptor VEGFR2 for use on tumor-associated endothelial cells that together have a key role in tumor neovascularization. Inhibitors of VEGF (e.g., bevacizumab) or VEGFR and its signaling pathway have been developed with the aim to target the pathological tumor endothelium and thereby cut off the blood supply to tumors and deprive cancer cells of nutrients and oxygen [142]. Besides the endothelial cells, VEGFRs can also be expressed on tumor cells, T cells, and TAM [143,144], and recent evidence suggests that inhibitors of VEGF and its receptors may exert important effects on tumor growth by affecting myeloid bystander cells [145]. In VEGFR2-expressing myeloid cells, VEGF was found to induce an immunosuppressive phenotype with upregulation of PD-L1, and VEGF blockade enhanced T cell activation, and potentiating the antitumor activity of the PD-1 checkpoint blockade [145]. VEGFR2 is also investigated as a CAR target, with the intention to eliminate the tumor-supportive vascular stroma of solid cancers [146,147]. Both alone and in combination with CAR T cells targeting tumor-cell associated antigens, VEGFR2-specific CAR T cells showed promising activity against tumors growing in mice. Our group found VEGFR2 expressed in human tumor biopsies of EwS and in EwS xenografts. VEGFR2-specific CAR T cells could be effective agents for modulating the TME in this cancer, making it an attractive combination partner for CAR T cells directed against tumor surface antigens such as G_D2_ [148] or B7H3 [149].

#### 5.1.3. Targeting the TME by Epigenetic Anticancer Agents

Insights into the epigenetic regulation of gene expression in myeloid cell populations could provide attractive targets for reprogramming these cells towards support, rather than suppression, of adaptive immune responses. Histone deacetylases have key roles in the priming of the proinflammatory M1 state of macrophage activation [53]. Additional effects of individual epigenetic agents in tumor cells and in T cells have to be taken into consideration. A selective inhibitor of class IIa HDAC, which affects gene expression in monocytes but not lymphocytes, was found to alter the TME by repolarizing TAM in a breast cancer mouse model, resulting in reduced tumor burden and metastasis, both alone and in synergy with PD-1 inhibitor therapy [150].

Another important regulator of macrophage activation is the enzymatic component of polycomb receptor complex 2 (PRC2), enhancer of zeste homolog 2 (EZH2) [151]. The blockade of EZH2 leads to the inhibition of proinflammatory pathways. Consequently, the EZH2 inhibitor tazemetostat, which was developed as an anticancer agent in the treatment of pediatric solid tumors, may also be effective to control inflammation in the TME. We recently found that EZH2 inhibitors reliably and selectively upregulate the pediatric CAR target G_D2_ in EwS [148]. Thus, inhibitors of EZH2 could be able to amplify the clinical activity of G_D2_-specific CAR T cells by overcoming both antigen escape and local immune suppression. However, controversial effects of EZH2 inhibitors on myeloid cell components in the tumor stroma were also reported. In an immune-competent mouse model, the EZH2 inhibitor GSK126 was found to increase the number of MDSC, while reducing antitumor effector cells, together masking the antitumor activity of GSK126 previously observed in immunodeficient models [152]. Controversial results obtained with epigenetic agents could be a consequence of dose-dependent mechanisms of action and on the variable composition of the TME in individual models. The clinical effects of these agents on the TME in humans and their capacity to boost antitumor T cell responses are difficult to predict.

### 5.2. Selective Targeting of Immunosuppressive Components of the TME

More selective interventions against immunosuppressive cells in the TME are developed for future combination, e.g., with T-cell based antitumor immunotherapies. One strategy is the blockade of chemoattractants and their receptors on myeloid suppressor cells, such as CXCR2 [121], CCL2 (MCP-1)/CCR2 [153], and CXCL12/CXCR4 [154]. The latter was found to promote T cell exclusion and suppression in murine models of metastatic breast cancer and is druggable by the clinically available agent plerixafor. Since numerous cytokines and multiple signaling networks are involved in the interaction between tumor cells and inflammatory cells in the TME, the relevance of an individual pathway will have to be clearly demonstrated before its therapeutic targeting.

Due to its high relevance for the tumor-permissive biology of TAM, the transmembrane tyrosine kinase colony-stimulating factor 1 receptor (CSF1R) is an attractive therapeutic target [46,155]. CSF1R is a key regulator of macrophage differentiation from their precursors, and it is critical for the recruitment and survival of TAM [46,156]. CSF1R signaling in TAM further promotes acquisition of immunosuppressive and pro-tumorigenic function, as well as resistance to the immune checkpoint blockade [157,158]. Antibody antagonists and small molecule inhibitors of CSF1R are currently developed as anticancer agents. CSF1R antagonists limit recruitment and immunosuppressive polarization of myeloid cells and directly deplete macrophages from the TME by apoptosis [62,159,160]. In mouse xenograft models of NBL and OS, myeloid cell depletion with an anti-murine CSF1R antagonist was highly effective to improve the efficacy of immunotherapy with a bispecific, T-cell engaging monoclonal antibody targeting G_D2_ [161]. Clinical CSF1R inhibition is an effective therapeutic principle in the treatment of a rare benign tumor, tenosynovial giant cell tumor (TGCT), which is characterized by aberrant CSF-1 secretion and the recruitment of CSF1R dependent inflammatory cells which dominate this tumor [162,163], and a small molecule CSF1R inhibitor, pexidartinib, has been approved for this indication. In malignant diseases, the efficacy of CSF1R inhibitors as single agents and even in combinations with cytotoxic or molecularly targeted agents, or with PD-1 checkpoint inhibitors, has so far been limited [164,165,166,167,168]. Understanding the mechanisms of failure may lead to more effective combination strategies. In glioblastoma mouse models, acquired resistance to CSF1R inhibition with rebounds of TAM was found to be mediated by the release of insulin-like growth factor (IGF), its interaction with IGF-1 receptor (IGF1R) on tumor cells and activation of protumorigenic downstream signals, providing a rationale for combined blockade of CSF1R with IGF1R and tumor tyrosine kinases [169].

A target with even higher selectivity for a relevant TAM subpopulation is the pattern-recognition scavenger receptor MARCO (macrophage receptor with collagenous structure). A non-depleting anti-MARCO antagonist reversed the immunosuppressive effects of TAM in murine tumors and enhanced the checkpoint therapy in melanoma and colon and lung carcinoma models [170,171]. Moreover, mannose receptor CD206 is selectively expressed on the M2-like TAM and can be targeted by an agent that induces a conformational switch to trigger apoptosis. This agent was found to both deplete and reprogram TAM, resulting in predominant M1 phenotypes, along with increased antitumor immune responses in both murine syngeneic and xenograft models of pancreatic cancer [172]. Finally, a CAR-based strategy was proposed to deplete MDSC via their expression of nonclassic MHC molecules acting as ligands for the activating NK-cell receptor NKG2D [173]. NK cells gene-modified to express a CAR composed of extracellular NKG2D fused to intracellular TCRζ signaling recognized and lysed human MDSC in vivo, including M-MDSC purified from human NBL, whereas T cells were spared, secreting T cell recruiting chemokines. The sequential treatment of NBL xenografts in mice with NKG2D-ζ expressing NK cells followed by G_D2_-specific CAR T cells resulted in elimination of MDSC from the TME, recruitment of CAR T cells into the TME, and improved antitumor activity.

### 5.3. Inflammatory Reprogramming of the TME Using Locoregional Cytokine or Oncolytic Virus Deposits

Innovative CAR T cell therapies aim to overcome barriers in the TME concurrent with the targeting of tumor cells. An all-in-one strategy proposed by Hinrich Abken´s group engineers T cells to express a CAR specific for a tumor-associated antigen, as well as a CAR-inducible transgene which encodes an immune-activating cytokine [174]. Such T cells redirected for universal cytokine killing (TRUCK) respond to target recognition, not only with a cytolytic T cell activation response, but also with release of the cytokine, e.g., IL-12, which can reprogram myeloid cells to lose their immunosuppressive properties. TRUCK with IL-12 secretion were found to be more effective than standard CAR T cells in tumor models, and this was associated with the recruitment of additional immune effector cells, like NK cells, and a reduction of MDSC [174,175,176,177]. More recently, the alternative cytokine IL-18 was used as the cytokine load of TRUCK and was found to promote the persistence of highly functional T cells, along with a proinflammatory TME [178,179,180,181]. G_D2_-specific CAR T cells with inducible IL-18 secretion are currently developed for pediatric cancers [182].

With a similar goal of promoting a supportive TME for therapeutic antitumor T cells, oncolytic viruses are promising partners for a synergistic combination strategy. Intratumoral administration of an oncolytic virus can create a proinflammatory TME, attracting CD8+ T cells, and thereby improving the efficacy of checkpoint inhibition [183,184]. Combinations with CAR T cell therapy in pediatric cancers are also pursued. An oncolytic adenovirus with natural tropism for the tumor was engineered to express a chemokine, CCL5, for optimal trafficking and a cytokine, IL-15, to sustain T cell proliferation, then combined with G_D2_-specific CAR T cells in a NBL model [185]. Combined therapy of the engineered oncolytic adenovirus and G_D2_-specific CAR T cells significantly enhanced survival compared to either of the two therapies alone.

All strategies aiming to increase the potency of CAR T cells must consider the immunological toxicities of current agents, such as cytokine release syndrome (CRS), immune cell-associated neurotoxicity (ICANS) [186], and tumor-inflammation associated neurotoxicity (TIAN) [187] that are a consequence of uncontrolled CAR T cell activation and expansion outside and within the CNS and that could become limiting in the presence of an enhancer.

### 5.4. Macrophage Immune Checkpoint Therapy: Unleashing TAM for Elimination of Tumor Cells

Rather than depleting TAM to enable effective adaptive antitumor immune responses, TAM can also be harnessed to exert their own antitumor effector function. Many cancer cells provide “eat me” signals for macrophage-mediated phagocytosis, e.g., calreticulin, but are simultaneously protected by expression of CD47, which engages SIRPα on macrophages and provides an antiphagocytic “don´t eat me” signal. CD47 or SIRPα blockade enables elimination of tumor cells, especially when combined with antitumor antibodies for macrophage-mediated cellular phagocytosis via the Fc domains of the bound antibody [188,189]. Several CD47 checkpoint inhibitors are in clinical development. As first clinical proof of evidence, a humanized anti-CD47 antagonist combined with rituximab showed activity in adult patients with non-Hodgkin lymphoma, including durable responses without dose-limiting toxicities [189].

Candidate diseases in pediatric oncology which express prophagocytic “eat me” signals are OS and NBL. CD47 and SIRPα are overexpressed in OS and correlate with higher CD163+ TAM infiltrates [107]. The blockade of CD47 on OS cells indeed reduced invasive ability and pulmonary metastasis in xenograft models, triggering phagocytosis [190]. The cytotoxic agent doxorubicin further induced calreticulin expression on the tumor cell surface and promoted macrophage-dependent phagocytosis in vitro [191]. Doxorubicin combined with CD47 antibody therapy exerted antitumor activity in an immunodeficient mouse model. In a syngeneic NBL model, lysis of tumor cells by a monoclonal antitumor antibody directed against O-acetylated G_D2_ was found to rely on the presence of macrophages, and a combination with SIRPα-specific monoclonal antibody enhanced the anti-NBL activity of O-acetylated G_D2_-specific antibody therapy [192].

Theruvath et al. recently reported strong synergy of anti-G_D2_ monoclonal antibody therapy with the CD47 blockade in various mouse models of NBL and OS, including orthotopic and metastatic tumor growth, and immunodeficient as well as syngeneic models [193]. Contrary to what was expected, the mechanism was not Fc-dependent. Instead, the anti-G_D2_ antibody disrupted the binding of G_D2_ to a newly identified ligand associated with inhibitory signaling on macrophages, Siglec-7, while upregulating calreticulin on the surface of the tumor cells. Together, this shifted the balance towards macrophage activation and phagocytosis.

Thus, the unleashing of potent innate immune responses by macrophages abundantly present in the TME of solid pediatric tumors could be a powerful strategy that is now being investigated in clinical studies.

### 5.5. CAR-Engineered Macrophages for Tumor-Targeted Phagocytosis

Macrophages, with their highly effective trafficking, infiltration into tissues, and preferential recruitment to tumors, as reflected by their especially high abundance in the typical solid tumors of childhood, are an attractive effector cell population as direct anticancer agents. Tumor targeting by macrophages could be achieved by the expression of CARs against tumor-associated surface antigens. Indeed, macrophages engineered to express an HER2-specific CAR with CD3ζ signaling were able to reduce tumor burden and enhance survival in mice exhibiting ovarian cancer xenografts [194]. The antitumor mechanism involved antigen-specific phagocytosis, along with the cross-presentation of tumor-associated antigens to CD8+ T cells. Macrophages expressing CARs were locked in a proinflammatory M1 state, even under M2-promoting culture conditions. Alternative CAR signaling domains other than T-cell derived TCRζ signaling could lead to even more potent CAR-engineered macrophage effectors. Moreover, CAR-modified macrophages could be attractive vehicles to deliver synergistic antitumor agents into the tumor niche by expressing additional transgenes.

## 6. Conclusions

Tumor cells in solid cancers grow and spread within networks of stroma containing various infiltrating immune cell populations. Multiparameter imaging of immune markers on tissue sections, as well as gene expression profiling, along with computational quantification algorithms now allow for the characterization of the cellular components of the TME. While comprehensive studies in pediatric solid tumors are still limited, largely consistent data from various groups demonstrate that the TME is dominated by myeloid cell components, with variable or little T cell infiltration. Together with the overall lack of response of pediatric cancers to the therapeutic principle of immune checkpoint blockade, along with experimental observations in mouse models, this suggests a significant barrier function of myeloid cell infiltrates against effective immune targeting. None of the available mouse models fully reflects the situation in humans. Therefore, studies of human tissues are needed to generate more substantial in vivo evidence and to characterize in detail the crosstalk between immune cell subpopulations and tumor cells in their individual niches. Pre- and post-therapeutic tumor tissues from patients treated in clinical immune therapy trials will provide the most valuable information.

From a translational point of view, a key issue is to identify and prioritize strategies to enable effective cellular immune therapy. Should myeloid cells be depleted, or should their polarization and function be modulated to allow CAR T cell infiltration into tumors and support their function? Which proinflammatory mediators are required to potentiate antitumor effector functions of T cells and stimulate (CAR) T cell memory? Simply unleashing macrophages to act as antibody-redirected effector cells for tumor cell lysis and phagocytosis could be a highly feasible option that does not require the manufacturing of complex and expensive cell products. The exceptional tissue penetration capacity of myeloid cells and their abundant natural recruitment for many pediatric solid cancers deserves attention, not only for use as barriers, but also for reengineering as novel antitumor effector cell populations.

## Data Availability

Not applicable.

## Abbreviations

5-FU	5-fluorouracil
ADCC	antibody dependent cell mediated cytotoxicity
ATRA	all-trans retinoic acid
c-FLIP	cellular FLICE-inhibitory protein
CAR	chimeric antigen receptor
CCL	C-C motif chemokine ligand
CCR	C-C chemokine receptor
CD	cluster of differentiation
COX2	cyclooxygenase 2
CSF1	colony stimulating factor 1
CSF1R	colony stimulating factor 1 receptor
CXCL	C-X-C motif chemokine ligand
CXCR	C-X-C motif chemokine receptor
EGF	epidermal growth factor
EwS	Ewing sarcoma
EWSR1-FLI1	Ewing sarcoma breakpoint region 1-friend leukemia integration 1
EZH2	enhancer of zeste homolog 2
Fc	fragment crystallizable
FLICE	FADD-like IL1-β-converting enzyme
GMR4	glutamate metabotropic receptor 4
HER-2	human epidermal growth factor receptor 2
HDAC	histone deacetylase
HLA-DR	human leukocyte antigen DR isotype
HSPC	hematopoietic stem and progenitor cell
IDO1	indoleamine 2,3-dioxygenase 1
IF	immunofluorescence
IFN-γ	interferon-γ
IGF	insulin-like growth factor
IGF1R	insulin-like growth factor receptor 1
IHC	immunohistochemistry
IL	interleukin
JAK	Janus kinase
LAG3	lymphocyte activating gene 3
MARCO	macrophage receptor with collagenous structure
MALDI-IMS	matrix-assisted laser desorption/ionization- imaging mass spectrometry
MCP1	monocyte chemoattractant protein-1
MCPcounter	microenvironment cell populations-counter
MDSC	myeloid-derived suppressor cells
MELC	multi-epitope-ligand cartography
MHC	major histocompatibility complex
M-MDSC	monocytic myeloid-derived suppressor cells
MMP9	matrix metalloproteinase 9
NA	non amplified
NBL	neuroblastoma
OS	osteosarcoma
P2 × 7R	P2X-Purinoreceptor 7
PD-1	programmed cell death protein 1
PD-L1	programmed cell death protein ligand 1
PI3Kγ	phosphatidyl-inositol 3 kinase γ isoform
PMN-MDSC	polymorphonuclear myeloid-derived suppressor cells
PRC2	polycomb receptor complex 2
RMS	rhabdomyosarcoma
RNAish	RNA in situ hybridization
ROS	reactive oxygen species
SIRPα	signal-regulatory protein alpha
STAT3	signal transducer and activator of transcription 3
TAM	tumor associated macrophages
TAN	tumor associated neutrophils
TCR	T-cell receptor
TGCT	tenosynovial giant cell tumor
TGF-β	transforming growth factor β
TLS	tertiary lymphoid structure
TME	tumor microenvironment
TNFα	tumor necrosis factor α
TRAIL	tumor necrosis factor-related apoptosis inducing ligand
TRUCK	T cells redirected for universal cytokine killing
UPS	undifferentiated polymorphic sarcoma
USP6	ubiquitin-specific protease 6
VEGF	vascular endothelial growth factor
VEGFR	vascular endothelial growth factor receptor
